# Performance‐Based Executive Functions Predict Internalising but Not Externalising Maladaptive Behaviour in Students With ID

**DOI:** 10.1111/jir.70027

**Published:** 2025-08-04

**Authors:** Stephan Kehl, Nina Römer

**Affiliations:** ^1^ Faculty of Participation Sciences Ludwigsburg University of Education Ludwigsburg Germany

**Keywords:** executive function, externalising and internalising behaviour, intellectual disability, maladaptive behaviour, mental health condition

## Abstract

**Background:**

Maladaptive behaviour is common in students with intellectual disability (ID). While executive functions (EFs) in typically developing children and adolescents are associated with maladaptive behaviour, there is currently contradictory and only fragmented empirical evidence on this association in students with ID. However, following impairments of EFs in this population, investigating this relationship could enhance the understanding of the development of maladaptive behaviour in students with ID.

**Method:**

The sample consisted of 45 students with ID (*M* = 11.8 years). Three core EFs (executive‐loaded working memory, switching, inhibition) were measured with performance‐based tasks, and maladaptive behaviour was assessed using a teacher report (BASC‐3).

**Results:**

Regression analyses showed that EF significantly predicted internalising but not externalising maladaptive behaviour. Specifically, working memory was positively related to internalising maladaptive behaviour. After controlling for fluid intelligence, age and sex, inhibition was negatively related to anxious maladaptive behaviour.

**Conclusions:**

This study yields valuable evidence on the relationship between performance‐based EF and maladaptive behaviour in students with ID and offers important implications for practice. Moreover, teachers should be aware that externalising maladaptive behaviour could be indicative of underlying mental health issues in students with ID, given the strong relationship between internalising and externalising maladaptive behaviour.

## 1 Introduction

According to the fifth revision of The Diagnostic and Statistical Manual of Mental Disorders (DSM), intellectual disability (ID) is characterised by both intellectual and adaptive functioning deficits in conceptual (e.g., reading, writing), social (e.g., social responsibility) and practical (e.g., self‐care, paying bills) domains. Moreover, the onset of intellectual and adaptive behaviour deficits must occur during the developmental period. Major changes with the latest revision of the DSM include the replacement of the term mental retardation and a stronger emphasis on adaptive functioning, what is now primarily used to determine the severity of ID and the level of support an individual requires (Pinchefsky and Shevell 2017).

Maladaptive behaviour of people with ID is an important limitation for their functionality in everyday life and a source of major concern for caregivers, teachers and parents (Rojahn et al. 2012). However, maladaptive behaviour is not a characteristic or domain of adaptive behaviour (Schalock et al. 2021), but according to Hartley et al. (2008), externalising (e.g., aggression or inattention) or internalising (e.g., depressive or anxious symptoms) behaviour problems that negatively influence activities of daily living. These problems can limit or exclude participation in social life (Royal College of Psychiatrists 2007). Maladaptive behaviour may be a manifestation or a symptom of psychiatric disorders, even though this link is empirically controversial (Rojahn and Meier 2009). In this study, maladaptive behaviour is conceptualised as mental health issues that, unlike psychiatric disorders, do not necessarily require a formal (medical) diagnosis. Empirically, they are commonly assessed via teacher and parents reports (Glasson et al. 2020).

Even though the prevalence of maladaptive behaviour in children and adolescents with ID is reported to be three to seven times higher than in typically developing children and adolescents (Alimovic 2013), moderators like severity of ID and aetiology influence the frequency and specificity of mental health issues. Some studies have yielded evidence of a higher frequency in people with lower intellectual functioning (Whitaker and Read 2006; White et al. 2005), even though others have found no significant differences (Einfeld and Tonge 1996; Whitney et al. 2019). With regard to aetiology, Glasson et al. (2020) found that people with Prader–Willi syndrome showed the highest rate of maladaptive behaviour, while prevalence was lowest for children and adolescents with Down syndrome (Di Nuovo and Buono 2011). However, the population of all studied genetic syndromes (including Fragile X and Williams syndrome) yielded higher prevalence rates than typically developing peers. Besides aetiology and severity of ID, prevalence varies according to the methodological design of the studies (e.g., the instrument used to identify mental health issues), the consideration of confounding variables (socio‐demographic and personal variables) and the specific mental health issue studied (e.g., internalising vs. externalising maladaptive behaviour).

## 2 Maladaptive Behaviour and Executive Functions (EFs)

EFs are mental top‐down processes. According to current empirical evidence, they are composed of three core components: inhibition/inhibitory control, executive‐loaded working memory/updating (WM) and switching/set shifting (Miyake et al. 2000; Miyake and Friedman 2012). Inhibition means the ability to suppress a dominant or habitual reaction and distractors (Diamond 2013; Miyake and Friedman 2012). WM stores information and processes it simultaneously (St Clair‐Thompson and Gathercole 2006). Switching allows cognitive flexibility to shift between tasks or mental sets (Diamond 2013; Miyake and Friedman 2012). Recent meta‐analyses have reported somewhat contradictory results regarding the functioning of EF in people with ID. Spaniol and Danielsson (2022) concluded that people with Williams syndrome and Fragile X syndrome had the most severe limitations in EF, while people with Down syndrome and non‐specific ID did not significantly differ from mental‐age matched controls. However, Tungate and Conners (2021) reported significantly lower EF in people with Down syndrome when compared with mental‐age matched controls.

A growing body of research supports the assumption that deficits in EF are generally associated with an increased risk of maladaptive behaviour (Snyder 2013; Snyder et al. 2015). Conceptionally, inhibitory control over one's behaviour, resisting temptations and not acting impulsively is one aspect of EF and closely linked to externalising maladaptive behaviour (Diamond 2013). Empirical evidence in typically developing children and adolescents supports this assumption (Hatoum et al. 2018; Riggs et al. 2003). Likewise, limitations in inhibitory control may increase the risk to develop internalising maladaptive behaviour through strengthening negative biases in attention and memory as well as limiting the capacity to suppress negative thoughts (Maasalo et al. 2021). Riggs et al. (2004) argue that casual to the relationship between inhibitory control and maladaptive behaviour is a potential separation of the current behaviour from future consequences. In accordance with the conceptual and empirical relation between inhibition and the other two core aspects of EF, empirical evidence mainly supports a common negative association between working memory and switching with maladaptive behaviour, even though the conceptual link may be less clear than with inhibitory control (Miyake and Friedman 2012; Snyder et al. 2015). However, in a clinical sample of children with ID and diagnosed mental health conditions, Santegoeds et al. (2022) found no consistent pattern of associations between performance‐based WM/inhibition and internalising/externalising maladaptive behaviour. Two studies examined the relationship between maladaptive behaviour and switching performance in people with ID and found no significant association between switching and externalising maladaptive behaviour, but with internalising maladaptive behaviour (Rockers et al. 2009; Visser et al. 2015). Based on observational reports, Frazier et al. (2022) reported that EF explained a very high proportion of variance (*R* ^2^ = 0.47) of externalising maladaptive behaviour in a diverse sample including children with developmental disability. Similar correlational results were reported by Esbensen et al. (2021) for teacher and parents' reports of EF and externalising maladaptive behaviour in children with Down syndrome. Gardiner and Iarocci (2018) also found a correlation between EF and internalising maladaptive behaviour in children with autism spectrum disorder without ID. However, the effect sizes were somewhat smaller in the last study.

## 3 The Present Study

To address the lack of consistency of empirical evidence, our first research objective is to analyse the reported prevalence of maladaptive behaviour in students depending on their fluid intelligence. Second, as the findings concerning the relationship between EF and maladaptive behaviour in students with ID are contradictory and fragmented, our second aim is to examine this relationship with performance‐based measurements of all three core components of EF. Finally, to detect the influence of EF on specific areas of maladaptive behaviour, we analyse their relationship with symptoms of aggression, anxiety, depression, hyperactivity and attention problems in students with ID while controlling for fluid intelligence, sex and age.

## 4 Method

### 4.1 Participants

An a priori power analysis yielded a required sample size of 45 for our main analysis, with an effect size of *f* ^2^ = 0.25 (two‐tailed) and a test power of 0.77 for a multiple regression. Even though effect sizes vary heavily, we chose a rather moderate to large estimate.

Participants were students (*N* = 45) from three special schools for children and adolescents with ID in Germany. Note that students in these schools are not necessarily diagnosed with ID according to the ICD‐10(11) or DSM because they are referred to these schools based on a special pedagogical (and not medical) assessment by special education teachers. Although there is no legal IQ criterion, in practice, students in these schools have severe learning problems and an IQ < 70/75.

The majority (61%) of the sample in our study were male students (see Table 1). While 82% of students were of non‐specific ID, 18% (*n* = 8) were reported to have Down syndrome. No other genetic syndrome was included. The mean age was 11.82 years (SD = 1.81) with a range of 7–18 years; fluid intelligence (Gf) ranged from 40 to 83 (*M* = 51.11, SD = 12.53). No other inclusion criteria (e.g., adaptive behaviour) for the sample were applied.

**TABLE 1:** Descriptive statistics (*N* = 45).

	*M* (SD)
Age	11.82 (1.81)
K‐ABC II Gf scale	51.11 (12.53)
	Maladaptive behaviour
Aggression	5.69 (4.56)
Anxiety	8.18 (3.96)
Depression	8.91 (4.92)
Hyperactivity	8.40 (5.50)
Attention problems	10.60 (4.47)
	Executive functions measurement
Working memory (WM)	9.62 (5.31)
Switching	20.27 (10.61)
Inhibition	1.96 (4.95)
Total EF score	41.84 (16.28)

### 4.2 Procedures

The study took place in urban and suburban areas in Germany and was approved by the ethical board of the University of Education Ludwigsburg on 12 June 2022 (application number III‐Sopaed‐StKe‐0016). Consent to participate was obtained prior to the beginning from all legal guardians and students. Students and parents were thoroughly informed with an information letter while the version for students was designed in accessible language.

Data collection on fluid intelligence and EF (Q1 2022–Q2 2023) was carried out by research assistants trained by the first and second author. Afterwards, teachers rated students' maladaptive behaviour via a standardised questionnaire.

### 4.3 Measures

#### 4.3.1 Maladaptive Behaviour

Maladaptive behaviour was measured using the German version of the Behaviour Assessment System for Children (teacher rating), Third Edition (BASC‐3) (Reynolds and Kamphaus 2015). The BASC‐3 is a one of the most frequently used tests to assess students' emotions and maladaptive behaviour (Canivez et al. 2021). Considering the discrepancy between the chronological and mental ages of students with ID, we used the standardised preschool questionnaires for teachers (Onnivello et al. 2022). In particular, many items in versions for school‐aged children and adolescences assessing anxious or depressive symptoms are heavily based on verbal skills of students (e.g., ‘Says, I hate myself’) and may not be sensitive enough for students with ID due to restrictions in expressive verbal abilities.

For our study, we included the subscales aggression (10 items, e.g., ‘Threatens to hurt others’), anxiety (nine items, e.g., ‘Is nervous’), attention problems (seven items, e.g., ‘Is easily distracted’), depression (nine items, e.g., ‘Is pessimistic’) and hyperactivity (nine items, e.g., ‘Has a low self‐control’). The items were rated by teachers on a 4‐point scale ranging from ‘never’ to ‘almost always’. Higher scores are indicative of more maladaptive behaviour. For further analyses, we used summed raw scores.

#### 4.3.2 Fluid Intelligence

As a measure of fluid intelligence, we used the subtests ‘story completion’ and ‘pattern reasoning’ from the KABC‐II to calculate a standardised score of general fluid intelligence (Gf) that we used for further analyses (Kaufman and Kaufman 2018). The KABC‐II is a comprehensive and well validated test battery for children and adolescents based on the CHC theory of intelligence (Schneider and McGrew 2018).

#### 4.3.3 EFs

We designed tests to measure the EF of students with IDs based on the recommendations of Snyder et al. (2015) and Henry and Winfield (2010) to minimise the task‐impurity problem. To motivate participants with ID, the assessment was based on a fictitious story about comic figures we called *mimis*. The participants' task was to help these figures or to play a game with them. All tasks were designed using Microsoft PowerPoint (2019) and presented on a 15‐inch screen of a Lenovo Yoga C940‐14IIL notebook.

**FIGURE 1:**
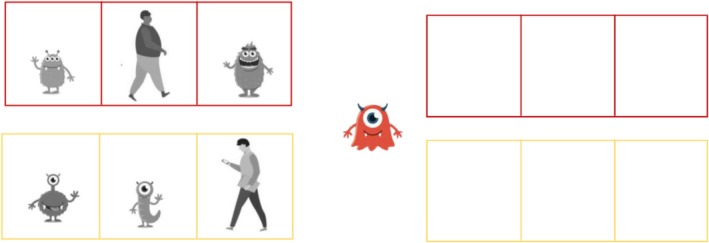
Adaption of the odd‐one‐out task with a list length of two. Participants had first to identify the human being in all rows and then, after they disappeared, had to point to the field where the figure was in the corresponding rows.

*WM* was assessed using an adaptation of the *odd‐one‐out task* (Henry 2012). Participants first had to identify a human being between two comic figures. Then, the figures disappeared, and the participants had to point to the field where the human was on the following slide (see Figure 1). The participants scored a point for each correctly identified field. Spans ranged from one to four, and the participants reached the next span when they performed at least three out of five tasks correctly.

**FIGURE 2:**
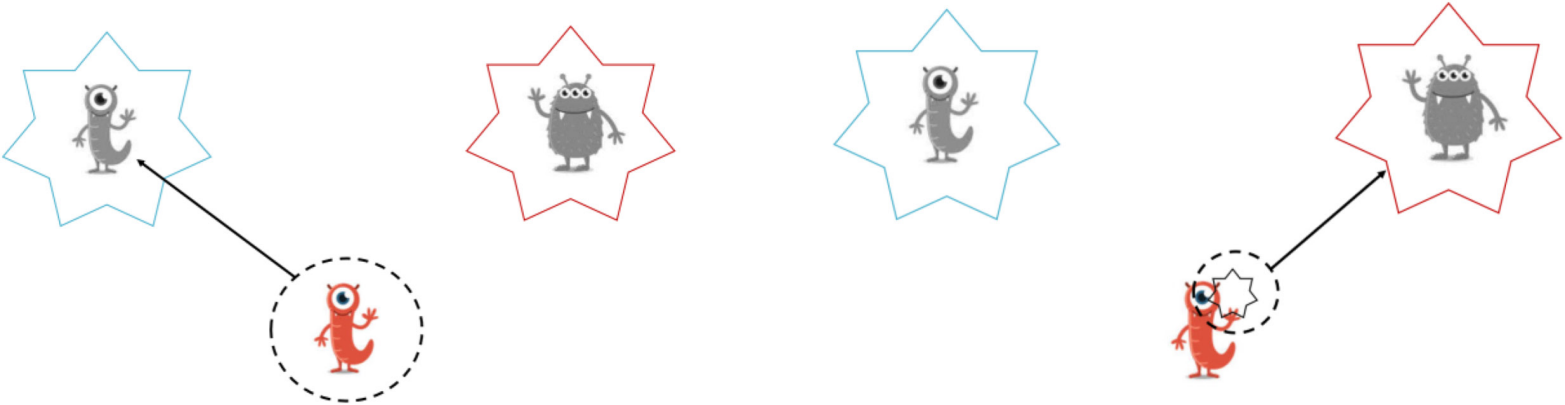
Instruction of the adaption of the DCCS. Students should first organise the comic figure according to its shape in the ‘mimi‐game’ (left), in the following game according to their colour (right). Finally, both conditions were mixed.

The *switching* component of EF was assessed using an adaption of the Dimensional Change Card Sort (DCCS) (Zelazo 2006). In the pre‐switching phase, the participants first played a shape game (‘mimi‐game’), where they were asked to arrange one comic figure according to its shape. If they performed the task correctly in at least five out of six trials, they moved on to the post‐switching phase, the colour game (‘star‐game’). Here, students were asked to arrange the comic figures according to their colour. If they performed the task correctly in at least five out of six trials, they proceeded to a phase where both conditions were mixed (‘border version’) (see Figure 2). To minimise working memory load, the instruction was repeated after every five trials in the boarder version. The difficulty was increased by covering the target stimulus with a distractor after 5 s in each condition. The instruction was repeated once if participants did not understand the task immediately. The raw scores were computed for the purpose of further analyses by summing up all correctly arranged figures.

The inhibition factor was assessed using an adapted version of the antisaccade task (Snyder et al. 2015). This task requires test persons to look in the opposite direction of a distractor to detect a briefly presented target stimulus. The recordings were obtained using a Tobii Pro Nano monitor–based eye tracker. A 9‐point calibration was performed to align the device. To ensure optimal recording, the distance between the subjects and the display had to be between 45 and 85 cm throughout the test. A tolerance range of 0.5° was applied in terms of accuracy and a 0.10° root mean square in terms of precision, based on the specifications of the developer (Tobii Pro AB 2022). If the values could not be achieved despite several recalibrations, the survey was still started. The quality of the collected data was checked during the data analysis.

**FIGURE 3:**
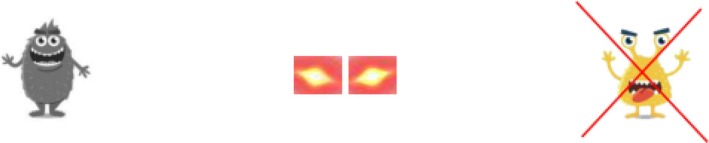
Instruction of the adaption of the antisaccade task. Students were instructed to fixate on the target (left) as fast as possible and not to look at the distractor (right).

In our adaption, the students were asked to prevent a comic figure from being scared by his friend by ‘beaming’ the figure away. The precise procedure was as follows: First, the students had to look at the middle of the screen to ‘load’ laser eyes for the game. This ensured that the starting point of fixation was the same across the trials (see Figure 3). Next, the students were asked to detect the target stimulus as fast as possible and to avoid looking at the distractor. After 1500–3500 ms, the distractor appeared on one side of the screen for 350 ms. Then, the target stimulus appeared on the other side of the screen. When participants visually fixated on the target stimulus, the experimenter clicked to the next slide. The difference between the number of fixations on the distractor item and the target item, each for a period of 1000 ms, was used as raw scores for further analyses.

In all three measures of EF, raw scores were used for further analyses and higher scores indicate a better performance.

## 5 Results

The statistical analyses were conducted using SPSS 29.0.0.0. The internal consistency (Cronbach's alpha) of maladaptive behaviour measured with the BASC‐3 was in an acceptable to excellent range: 0.87 for aggression, 0.76 for anxiety, 0.89 for depression, 0.89 for hyperactivity and 0.92 for attention problems. The composite score of internalising maladaptive behaviour (*α* = 0.90) and externalising maladaptive behaviour (*α* = 0.94) yielded a slightly higher internal consistency.

For our measures of EF, the internal consistency was generally lower but in line with the findings of other research (Poloczek et al. 2016) ranging from 0.57 (WM) and 0.74 (switching) to 0.92 (inhibition). The internal consistency of the fluid intelligence scale was rather low, too (0.65). However, validity was supported by significant correlations of all EF measures with the raw scores for fluid intelligence in the expected direction with *βs* from 0.29 (inhibition) to 0.82 (WM); all *p*s < 0.041 (one‐tailed).

Participants with missing data (e.g., students not able to complete the assessment) were excluded prior to the statistical analyses; therefore, the total sample was used for further data processing (*N* = 45). The descriptive results are shown in Table 1.

To analyse whether maladaptive behaviour differed according to the fluid intelligence of the participants, we computed Spearman's rank correlation coefficients with bias‐corrected and accelerated (BCa) 95% confidence intervals. Both internalising domains (anxiety and depression) significantly correlated with fluid intelligence, *r* = 0.34, *p* = 0.025, 95% BCa [0.03; 0.59] and *r* = 0.30, *p* = 0.044, 95% BCa [0.03; 0.54], respectively. Conversely, no externalising maladaptive behaviour was significantly associated with fluid intelligence, all *r*s < 0.21, all *p*s > 0.165.

To control for fluid intelligence, age and sex in our correlational analysis of EF and maladaptive behaviour, we ran a Pearson's correlation for the total sample (*N* = 45) with accelerated and bias‐corrected 95% confidence intervals for the bivariate variables.

The results of the partial correlational analysis are presented in Table 2. No significant correlations were found for any of the EF and maladaptive behaviour. In contrast, almost all areas of maladaptive behaviour were significantly intercorrelated with each other, all *r*s > 0.30, all *p*s < 0.056.

**TABLE 2:** Partial correlations adjusted for fluid intelligence, age and sex for the total sample (*N* = 45).

	1	2	3	4	5	6	7	8	9	10
1. Working memory (WM)										
2. Switching	**0.58** **									
3. Inhibition	−0.02	0.02								
4. Executive functions (total score)	**0.69** **	**0.92** **	**0.37** *							
5. Aggression	0.18	0.19	−0.09	0.15						
6. Anxiety	0.27	0.27	−0.30	0.17	**0.47** *					
7. Depression	0.21	0.15	−0.22	0.09	**0.62** **	**0.72** **				
8. Hyperactivity	0.11	0.15	−0.20	0.07	**0.73** **	**0.62** **	**0.79** **			
9. Attention problems	−0.13	−0.09	−0.10	−0.14	**0.47** *	**0.30**	**0.53** **	**0.62** **		
10. Internal maladaptive behaviour	0.26	0.22	−0.27	0.14	**0.60** **	**0.91** **	**0.94** **	**0.77** **	**0.46** *	
11. External maladaptive behaviour	0.06	0.10	−0.16	0.04	**0.84** **	**0.55** **	**0.76** **	**0.92** **	**0.80** **	**0.72** **

* *p* < 0.05.

** *p* < 0.001.

*Note:* Statistically significant *p*‐values (< 0.05) are in bold.

For our main analysis, we first computed two regression models with a composite score of internalising maladaptive behaviour (*α* = 0.90) and externalising maladaptive behaviour (*α* = 0.94) for the total sample (*N* = 45). The predictors were the EF. A visual inspection of the data via a histogram, a P–P plot, and a scatterplot of the standardised predicted values suggested a potential violation of the assumptions of the normality of residuals and homoscedasticity. Therefore, BCa bootstrap *p*‐values and 95% confidence intervals for unstandardised regression coefficients were computed. In contrast, the analysis of collinearity (VIFs < 1.77) and influential cases (Cook's distances < 0.17) did not give reason for concern.

The EF explained a significant proportion of the variance in internalising maladaptive behaviour, *R* ^2^ = 0.21, *p* = 0.023, but not in externalising maladaptive behaviour, *R* ^2^ = 0.05, *p* = 0.537. WM significantly predicted internalising maladaptive behaviour, *β* = 0.38, *p* = 0.045.

Second, we conducted several multiple regressions with aggression, anxiety, depression, hyperactivity, and attention problems as outcome variables and sex, age, and fluid intelligence as control variables. The predictors were put into the model simultaneously (forced entry). BCa bootstrap *p*‐values and 95% confidence intervals of unstandardised regression coefficients were calculated to address the potential violation of homoscedasticity and the normality of residuals. The analysis of influential cases (Cook's distances < 0.32) indicated no reason for concern. Following the high correlation of WM and fluid intelligence, there was some multicollinearity in the regression model, but still of an acceptable size (VIFs for WM and fluid intelligence < 4.2). However, the explained variance was possibly underestimated and the accuracy of these coefficients limited.

The change of explained variance was only substantial for anxiety, Δ*R* ^2^ = 0.16, *p* = 0.051 (see Table 3). Inhibition significantly predicted anxiety, *β* = −0.30, *p* = 0.030. No other regression model or predictor was significant. For all areas of externalising maladaptive behaviour (aggression, hyperactivity, attention problems), the change in explained variance varied between 0.03 and 0.06; all *p*s > 0.462. All predictors were smaller than *β* = 0.22 and not significant (all *p*s > 0.201).

**TABLE 3:** Linear models of predictors of anxiety (*N* = 45), 95% BCa confidence intervals based on 1000 bootstrap samples are reported in parentheses.

	*b*	SE	*β*	*p*
Model 1				
Sex	2.29 [−0.33, 4.87]	1.20	0.29	0.059
Age	0.15 [−0.37, 0.87]	0.32	0.07	0.604
Fluid intelligence	0.12 [0.03, 0.21]	0.05	0.36	0.009
Model 2				
Sex	2.54 [−0.53, 5.42]	1.20	0.32	0.068
Age	0.03 [−0.52, 0.65]	0.30	0.01	0.931
Fluid intelligence	0.06 [−0.09, 0.19]	0.08	0.18	0.371
Working memory (WM)	0.19 [−0.23, −0.67]	0.21	0.25	0.379
Switching	0.07 [−0.06, 0.21]	0.07	0.18	0.340
Inhibition	−0.24 [−0.47, −0.01]	0.12	−0.30	0.030

*Note:* *R* ^2^ = 0.15 for Model 1 (*p* = 0.077), Δ*R* ^2^ = 0.16 for Model 2 (*p* = 0.051).

## 6 Discussion

In the current study, we examined the relationship between performance‐based EF and teacher reports of internalising/externalising maladaptive behaviour in students with ID.

Concerning our first research question, we found that anxiety and depression were positively associated with fluid intelligence, while there was no significant correlation with externalising maladaptive behaviour and fluid intelligence. This contradicts the findings of some previous studies (Einfeld and Tonge 1996; Maïano et al. 2018; Whitney et al. 2019) but is in line with the findings of a recent meta‐analysis by Maïano et al. (2018) who reported that students with higher intellectual functioning were at higher risk of a depression disorder. One reason for this may be that teachers fail to recognise symptoms of anxiety or depression in students with more severe forms of ID due to diagnostic overshadowing (Platt et al. 2019). Additionally, our questionnaire may not have allowed identification of atypical symptoms in students with more severe ID (Hurley 2008). Alternatively, internal factors may contribute to that difference because of the relationship between cognitive development and self‐concept (Glenn and Cunningham 2001).

With regard to our second research question, EF similarly predicted internalising maladaptive behaviour, but did not predict externalising maladaptive behaviour. This finding partially contradicts previous empirical evidence on students with developmental disabilities (Esbensen et al. 2021; Frazier et al. 2022; Santegoeds et al. 2022) and without developmental disabilities (Halse et al. 2022). Methodological differences in assessing EF (Frazier et al. 2022) or peculiarities of the sample (Rockers et al. 2009; Santegoeds et al. 2022) may contribute to this disparity. WM (*β* = 0.38) was a significant predictor of internalising maladaptive behaviour, which could mirror differences in general cognitive capacity, given the strong relationship of WM and fluid intelligence in our study. General cognitive development may positively relate to symptoms of anxiety because students with rather high cognitive functioning can express their emotions more precisely, therefore facilitating identification. Alternatively, better WM in students with ID could facilitate the maintenance of negative thoughts and worries, possibly resulting in more anxious or depressive maladaptive behaviour.

Inhibition significantly predicted anxiety when controlling for fluid intelligence, age and sex. This finding is in line with empirical evidence (Hughes and Ensor 2011; Riggs et al. 2003). Inhibition might be generally negatively related to anxiety because of its influence on emotion regulation or difficulties in suppressing negative feelings and cognitions (Hatoum et al. 2018). This relationship may indicate an association between mental‐age matched difficulties in inhibitory control in students with ID (Lifshitz et al. 2016; Spaniol and Danielsson 2022) and higher rates of anxious symptoms in this population (Platt et al. 2019; Whitaker and Read 2006; White et al. 2005). However, not all studies support this assumption (Maïano et al. 2018), so inferences must be drawn cautiously. Finally, the non‐significant association between switching and externalising maladaptive behaviour in our study is aligned with the findings of Visser et al. (2015) and Rockers et al. (2009). However, our findings do not support an association of switching with internalising maladaptive behaviour. We hypothesise that the conclusions of Rockers et al. (2009) may be due to their study's specific sample of adolescents and young adults with 22q11.2 deletion syndrome and/or its correlational design that did not control for other cognitive variables or other EF.

Notwithstanding the non‐significant correlations between EF and maladaptive behaviour, the statistical pattern of our results descriptively corresponds fairly well to that of Hatoum et al. (2018), who investigated this relationship in children and adolescents without ID. Generally, maladaptive behaviour is negatively related to inhibition but positively to WM and switching. The positive/negative relationship of switching and inhibition with maladaptive behaviour may reflect a stability‐flexibility trade‐off, because less interference from irrelevant goals may enhance switching (Hatoum et al. 2018; Herd et al. 2014). Conversely, the nature of the positive relation of maladaptive behaviour with WM is still unclear and needs further empirical and theoretical clarification.

Our results have important practical implications. The lack of association of EF and fluid intelligence with externalising maladaptive behaviour may point to the impact of educational practices and the social environment. Indeed, challenging behaviour among persons with intellectual disabilities can be effectively reduced with different strategies including contextual interventions (Heyvaert et al. 2010). However, as we did not systematically study this relationship, this hypothesis needs to be addressed in future research. Second, given the strong relationship between internalising and externalising maladaptive behaviour in students with ID, a child with ID exhibiting externalising symptoms may be at higher risk of developing internalising symptoms following social isolation or rejection (Hatoum et al. 2018). Moreover, teachers must be aware that externalising behavioural problems can also be indicative of a mental health condition because symptoms may differ between people with and without ID (e.g., Platt et al. 2019). Hence, in addition to strategies of behavioural modification or contextual interventions addressing problematic behaviour, students may need more psychological and/or educational support because of potential underlying mental health issues. Particularly, internalising mental health issues in students with moderate ID may often be overseen following diagnostic overshadowing. Finally, given the influence of EF on anxiety symptoms, interventions tackling EF may also positively influence anxious maladaptive behaviour. The combination of physical activity with personality development, like in traditional martial arts, or mindfulness strategies could be an effective way of addressing inhibitory control and, therefore, internalising maladaptive behaviour (Diamond 2013; Esbensen et al. 2021).

Alongside these important practical implications, our study has several limitations. First, the sample size is small, and our study has low power with regard to the correlational analysis, where a higher power might have contributed to more significant results. Due to an expected moderate to large effect size, we probably underestimated the necessary size of our sample. Second, the external validity of our study is not clear, as the sample is not representative of students with ID. Comparing our sample with a representative study of students in schools for children and adolescents with ID in another German federal state (Bavaria), we had a similar share of male students (61%) and students without a specific genetic syndrome (82%). However, our sample showed a slightly higher proportion of students with DS (18% vs. 13%), while no other genetic syndromes were included (Baumann et al. 2021). Moreover, as students had to perform performance‐based measures of EF and fluid intelligence, children and adolescents with more severe limitations in intellectual functioning were not part of the study. Finally, we did not control for potentially different mechanisms between EF and maladaptive behaviour according to aetiology, because our objective was to reveal general links between EF and maladaptive behaviour in students with ID.

As we studied students in special schools for children and adolescents with ID, they do not necessarily have a medical diagnosis of ID according to the criteria of the DSM or ICD (intellectual and adaptive functioning) because placement is based on special pedagogical assessment. We did not assess adaptive functioning, language skills or other cognitive variables that may influence the association between EF and maladaptive behaviour.

A limiting factor of our methodology is that maladaptive behaviour was not measured in a multi‐perspective way. For instance, Ruddick et al. (2015) and Emerson et al. (2005) showed that primary caretakers rate the personal needs as well as behavioural and emotional problems of children with ID higher than teachers. Conversely, Esbensen et al. (2021) concluded that teachers report higher rates of concern in maladaptive behaviour, which could be due to the fact that different settings (home vs. school) put different demands on children's behaviour and EF (Onnivello et al. 2022). Empirical evidence indicates that teachers and parents ratings may particularly deviate from each other when assessing internalising (vs. externalising) maladaptive behaviour (Stanger and Lewis 1993). Children may show depressive or anxious symptoms in the presence of their parents, but not teachers. The association of EF, fluid intelligence and maladaptive behaviour as well as the frequency of maladaptive behaviour may, therefore, differ when including parents' reports. Moreover, one should keep in mind that we used the preschool version of the BASC for assessing maladaptive behaviour in students with a mean age of around 12. Even though that reduced the verbal load of items, there may be a deficiency in identifying symptoms of maladaptive behaviour typically for older students and/or students with a higher level of intellectual functioning.

A second point regarding our instruments concerns the measure of EF. As we designed new instruments to assess EF, there is limited support for the reliability and validity of these measures. In particular, the low internal consistency of WM gives reason for concern. Likewise, internal consistency for the standardised scale of fluid intelligence was rather low, too.

Finally, as our study is correlational in nature, the precise link between EF and maladaptive behaviour is still under question. Higher rates of maladaptive behaviour may contribute to limited EF, or vice versa (Donati et al. 2021).

## Ethics Statement

The study was approved by the ethical board at the University of Education Ludwigsburg (application number III‐Sopaed‐StKe‐0016) and conducted in accordance with the Declaration of Helsinki. Consent to participate was obtained prior to the beginning from all legal guardians and students.

## Conflicts of Interest

The authors declare no conflicts of interest.

## Data Availability

The data that support the findings of this study are available on request from the corresponding author. The data are not publicly available because of privacy or ethical restrictions.
